# Alpelisib in PIK3CA-Related Overgrowth Spectrum (PROS): A Systematic Review of Real-World Evidence in over 100 Patients

**DOI:** 10.3390/cells15090788

**Published:** 2026-04-27

**Authors:** Francesco Pellegrino, Giuseppe Reynolds, Simona Cardaropoli, Maria Luca, Stefania Massuras, Diana Carli, Alessandro Mussa

**Affiliations:** 1 Department of Public Healthcare and Pediatrics, University of Torino, 10124 Torino, Italy; giuseppe.reynolds@unito.it (G.R.); simona.cardaropoli@unito.it (S.C.); stefania.massuras@unito.it (S.M.); alessandro.mussa@unito.it (A.M.); 2 Neonatal Special Unit, Regina Margherita Children Hospital, 10126 Torino, Italy; 3 Postgraduate School of Pediatrics, Department of Public Health and Pediatrics, University of Torino, 10124 Torino, Italy; 4 Pediatric Clinical Genetics Unit, Regina Margherita Children Hospital, 10126 Torino, Italy; maria.luca@unito.it (M.L.); diana.carli@unito.it (D.C.); 5 Department of Medical Sciences, University of Torino, 10124 Torino, Italy

**Keywords:** *PIK3CA*-related overgrowth spectrum, PROS, PI3K pathway, alpelisib, targeted therapy

## Abstract

**Background**: *PIK3CA*-related overgrowth spectrum (PROS) comprises a heterogeneous group of mosaic disorders caused by activating variants in the *PIK3CA* gene, resulting in dysregulation of the PI3K/AKT/mTOR signaling pathway and abnormal tissue overgrowth. Targeted inhibition of this pathway has recently emerged as a promising therapeutic strategy. **Methods**: We conducted a literature review to identify published reports describing patients with PROS treated with alpelisib, a selective inhibitor of the p110α catalytic subunit of PI3K. Data regarding patient characteristics, genetic variants, treatment regimens, clinical outcomes, radiological response, and adverse events were extracted and analyzed. **Results**: Seventeen publications met the inclusion criteria, comprising a total of 114 patients treated with alpelisib. The majority of patients were pediatric (68.4%), with a median age at treatment initiation of 12 years. Clinical manifestations were heterogeneous and included segmental overgrowth, vascular malformations, and soft-tissue hypertrophy. Clinical improvement in at least one disease manifestation was reported in 111 patients (97.3%). Radiological response, defined as reduction ≥20% in lesion volume, was documented in 26 of 60 evaluable cases (47.3%). Adverse events were reported in 64 patients (56.1%) and were generally mild and manageable, with hyperglycemia and diarrhea being the most common. **Conclusions**: Available real-world evidence suggests that alpelisib provides meaningful clinical benefit across multiple PROS phenotypes, with an acceptable safety profile. However, current data remain limited by small cohort sizes, heterogeneous outcome reporting, and variable follow-up duration. Prospective studies with standardized outcome measures are needed to better define long-term efficacy and safety of PI3K inhibition in PROS.

## 1. Introduction

*PIK3CA*-related overgrowth spectrum (PROS) comprises a heterogeneous group of rare developmental disorders characterized by asymmetric and disproportionate tissue overgrowth caused by post-zygotic activating variants in the *PIK3CA* gene [1].

These mutations arise after fertilization and occur in a mosaic pattern, leading to the coexistence of genetically distinct cell populations and resulting in segmental, tissue-specific disease manifestations.

PROS encompasses a spectrum of overlapping entities, including congenital lipomatous overgrowth, vascular malformations, epidermal nevi, and skeletal anomalies (CLOVES), megalencephaly–capillary malformation syndrome (MCAP), Klippel–Trenaunay syndrome (KTS), and fibroadipose overgrowth, which are increasingly recognized as part of a continuous phenotypic spectrum rather than distinct disorders.

The clinical presentation is highly variable and may include adipose and soft-tissue overgrowth, vascular and lymphatic malformations, skeletal abnormalities, and neurological involvement. This phenotypic heterogeneity and the severity are primarily determined by the timing of the postzygotic mutational event, the degree of mosaicism, and the distribution of affected cell populations across tissues [2,3,4,5].

In spite of an estimated incidence approaching 1:22.313 individuals [6] and its considerable contribution to lateralized overgrowth [7,8,9], the disease is likely underestimated, given its phenotypic heterogeneity and challenges in molecular diagnosis.

**Figure 1:**
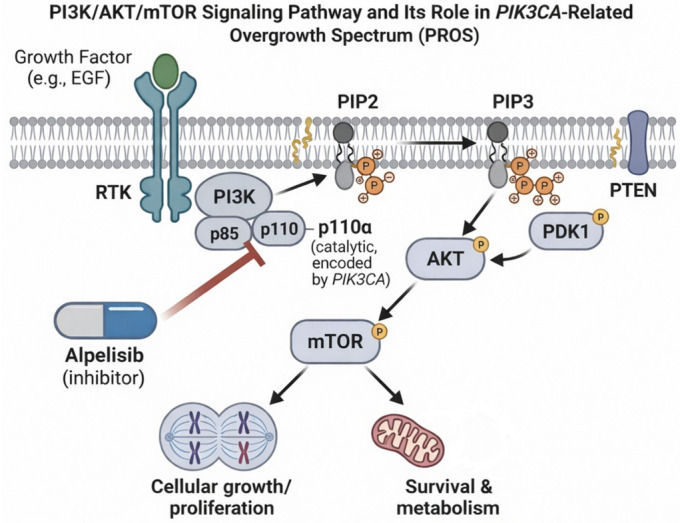
PI3K/AKT/mTOR signaling pathway and therapeutic targeting in *PIK3CA*-related overgrowth spectrum (PROS). Schematic overview of the PI3K/AKT/mTOR signaling cascade. Activation of receptor tyrosine kinases stimulates PI3K, composed of the regulatory subunit p85 and the catalytic subunit p110α encoded by *PIK3CA*, leading to conversion of PIP2 to PIP3 and downstream activation of AKT and mTOR signaling. This pathway regulates cellular growth, proliferation, survival, and metabolism. In PROS, activating *PIK3CA* variants result in constitutive pathway activation and tissue overgrowth. Alpelisib selectively inhibits the p110α catalytic subunit, thereby suppressing PI3K signaling and providing a targeted therapeutic strategy.

At the biological level, PROS is driven by overregulation of the phosphoinositide 3-kinase (PI3K)/AKT/mTOR signaling pathway, a key controller of cell growth, metabolism, and survival. *PIK3CA* encodes the catalytic p110α subunit of class I PI3K, which normally interacts with regulatory subunits that maintain the enzyme in an inactive state. Upon activation, PI3K converts phosphatidylinositol-4,5-bisphosphate (PIP2) into phosphatidylinositol-3,4,5-trisphosphate (PIP3), triggering downstream the activation of AKT and, ultimately, the mechanistic target of rapamycin complex 1 (mTOR) [6] (Figure 1).

**Figure 2:**
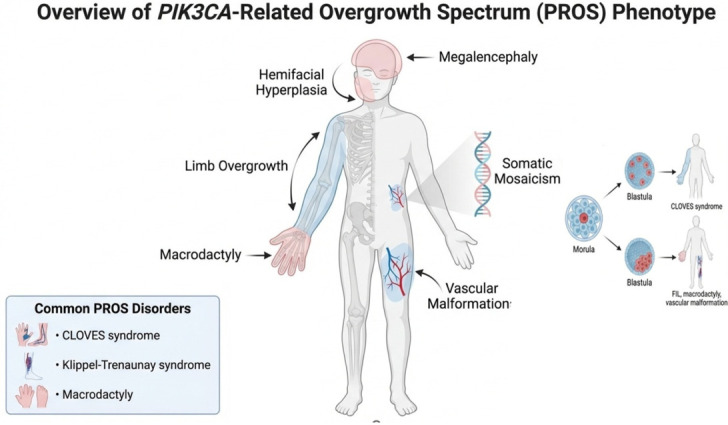
Clinical overview of *PIK3CA*-related overgrowth spectrum (PROS). Schematic representation of the phenotypic and molecular features associated with PROS. Somatic activating variants in *PIK3CA* lead to dysregulation of the PI3K/AKT/mTOR signaling pathway, resulting in mosaic abnormal cellular proliferation, tissue overgrowth, and malformations. The clinical manifestations are highly heterogeneous and may include segmental limb overgrowth, macrodactyly, hemi-hyperplasia (lateralized overgrowth), (hemi-)megalencephaly, and complex vascular anomalies. PROS encompasses a spectrum of disorders characterized by variable distribution and severity of tissue involvement, including several conditions defined as separated syndromes (e.g., congenital lipomatous overgrowth, vascular malformations, epidermal nevi, and skeletal anomalies—CLOVES—or Klippel–Trenaunay syndromes—KTS) but representing a continuum (spectrum). Phenotypic heterogeneity arises from both the anatomical distribution of mutant cells and the magnitude of overgrowth, reflecting tissue-specific mosaicism and the combined effect of variant type and mutant cell burden.

Persistent activation of this signaling cascade promotes abnormal cellular proliferation and, finally, drives overgrowth of a variable combination of multiple tissue types, including adipose, vascular, connective, skeletal, and neural ones. Phenotypic diversity in PROS arises from both the anatomical distribution of mutant cells and the magnitude of overgrowth, reflecting tissue-specific mosaicism and the combined effect of variant type and mutant cell burden [10,11] (Figure 2).

Historically, management of PROS has been largely symptomatic, relying on repeated combinations of surgical procedures (including debulking, osteotomies, epiphysiodesis and limb-length equalization) and interventional radiological techniques such as sclerotherapy or embolization for vascular malformations, approaches that often require multiple interventions over time and do not address the underlying molecular driver of the disease. While these approaches may alleviate specific complications, they usually deliver only partial or temporary benefit and may be associated with considerable morbidity [12]. The recognition that PROS is driven by activating variants in the PI3K–AKT–mTOR pathway has paved the way for targeted therapies, with PI3Kα inhibitors such as alpelisib emerging as promising disease-modifying treatments capable of directly counteracting the underlying molecular mechanism. These advances mark a paradigm shift in the management of PROS, moving from predominantly palliative interventions toward mechanism-based therapies that directly target the molecular driver of the disorder and hold promise for more durable disease control. Accordingly, multiple pharmacological approaches have been investigated to inhibit the PI3K–AKT–mTOR signaling cascade at different nodes along the pathway. Early therapeutic efforts focused primarily on mTOR inhibitors, such as sirolimus, which has been widely used as a therapy for vascular anomalies, although it demonstrated partial clinical benefit in some patients but variable and often incomplete responses [13,14,15,16,17]. More recently, attention has shifted toward inhibitors acting upstream in the pathway, particularly PI3Kα inhibitors, with alpelisib emerging as a targeted therapy specifically designed to counteract the hyperactivation of PI3K signaling that characterizes PROS. Alpelisib (BYL719) is an orally available selective inhibitor of the p110α catalytic subunit encoded by *PIK3CA*. Initially developed for the treatment of cancers harboring activating *PIK3CA* mutations [18], it has recently emerged as a promising therapeutic option for patients with PROS by directly targeting the dysregulated signaling pathway responsible for abnormal tissue growth [12]. Clinical evidence supporting the efficacy of PI3K inhibition in PROS has been provided by the EPIK-P1 study conducted by Canaud and colleagues, which demonstrated significant clinical improvements in patients with severe disease treated with alpelisib [19,20,21]. Additional evidence on the therapeutic role of PI3K inhibition in PROS is being generated through ongoing clinical trials, including EPIK-P2, EPIK-P3 and EPIK-P4, designed to further evaluate the efficacy and safety of alpelisib across different clinical contexts. Notably, the results of the EPIK-P2 study supported the regulatory approval of alpelisib in the United States for the treatment of patients aged ≥ 2 years with severe manifestations of PROS requiring systemic therapy.

Beyond these ongoing trials, an increasing number of case reports and small clinical series have described the use of alpelisib in patients managed in routine clinical practice. These observations are particularly valuable, as they provide insight into the performance of targeted therapies in heterogeneous patient populations outside controlled trial settings. Importantly, patients treated in routine clinical practice may differ from those included in EPIK-P1 in terms of selection criteria, baseline disease characteristics and outcomes, underscoring the value of real-world evidence for assessing the external validity of trial results.

In this review, we summarize the currently available literature on alpelisib treatment in PROS, focusing on evidence derived from clinical practice in order to better characterize therapeutic outcomes and highlight current limitations, including the need for more standardized and homogeneous follow-up data.

## 2. Materials and Methods

### 2.1. Literature Search Strategy

A systematic literature search was conducted to identify published reports describing patients with PROS treated with alpelisib. Electronic databases, including PubMed, Scopus, and Web of Science, were searched from database inception to December 2025. The search strategy combined terms related to the disease and treatment, including “*PIK3CA*-related overgrowth spectrum”, “PROS”, “*PIK3CA* mutation”, “overgrowth syndrome”, and “alpelisib” or “BYL719”. Additional articles were identified through manual screening of reference lists of relevant publications and review articles.

### 2.2. Eligibility Criteria and Studies Selection

Studies were considered eligible if they met the following inclusion criteria:

1. Reported patients diagnosed with PROS or related conditions caused by activating *PIK3CA* variants.
2. Patients were treated with alpelisib.
3. Provided clinical information regarding patient characteristics, treatment indication, or outcomes.

**Figure 3:**
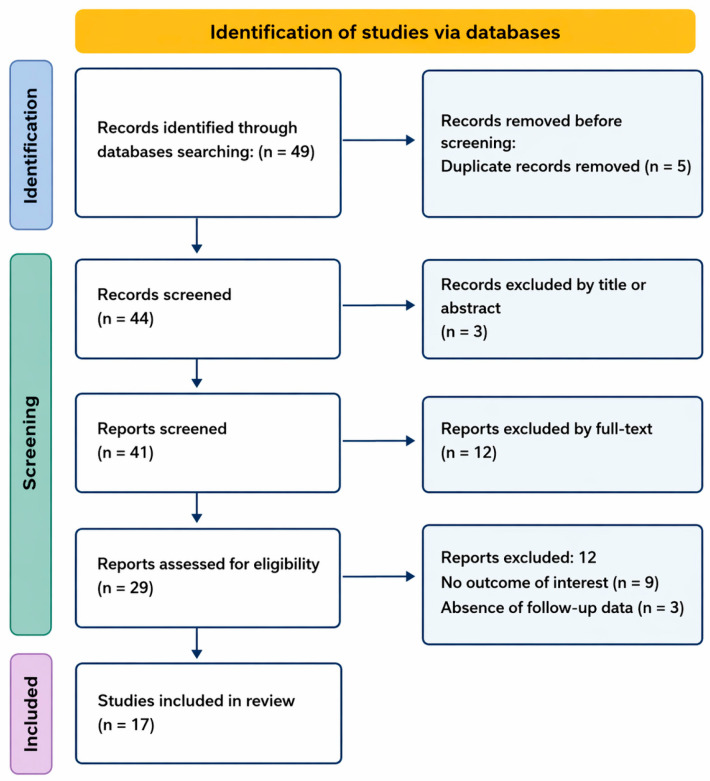
PRISMA flow diagram of study selection.

Case reports, case series, and observational studies were included. Clinical trial, animal studies and conference abstracts without sufficient clinical information were excluded. All records identified through database searches were screened for eligibility in two stages. First, titles and abstracts were reviewed to exclude clearly irrelevant publications. Subsequently, the full text of potentially eligible articles was assessed for inclusion based on the predefined criteria. When multiple publications reported overlapping patient cohorts, the most complete dataset was included to avoid duplication. A formal protocol for this systematic review was not prospectively registered (e.g., in PROSPERO). The study selection process followed a PRISMA-inspired approach [22], with predefined eligibility criteria and a structured data extraction process to ensure methodological transparency and reproducibility. The screening procedure is summarized in Figure 3.

### 2.3. Data Extraction and Analysis

Data from eligible studies were extracted using a standardized data collection form. The following variables were recorded when available:

- Patient demographic characteristics (sex and age);
- Clinical phenotype and affected tissues;
- *PIK3CA* variants;
- Medical history;
- Treatment regimen (age at start, starting dose and duration);
- Clinical and radiological response to therapy;
- Reported adverse events;
- Follow-up duration.

When studies reported multiple patients, data were extracted individually for each case whenever possible. Given the heterogeneity of study designs and outcome measures across the included reports, a descriptive analysis was performed. Patient characteristics, genetic variants, treatment indications, and clinical outcomes were summarized using descriptive statistics. When appropriate, categorical variables were presented as counts and percentages, while continuous variables were summarized as medians and ranges. The differences between groups were analyzed with Fisher’s exact test for categoric data. The significance was set at *p* < 0.05. Adverse events were graded according to the Common Terminology Criteria for Adverse Events (CTCAE), with grades ranging from mild (grade 1) to severe (grade 3), while serious adverse events were defined based on regulatory criteria, including events resulting in hospitalization, life-threatening conditions, or significant disability.

Flow diagram illustrating the process of study identification, screening, eligibility assessment, and inclusion for the present review.

## 3. Results

### 3.1. Study Selection

The literature search identified a total of 49 records from electronic databases and additional sources. After removal of duplicates, 44 records were screened based on title and abstract. Following this screening step, 29 full-text articles were assessed for eligibility. A total of 17 publications met the inclusion criteria and were included in the final analysis, comprising a total of 114 individual patients treated with alpelisib [19,23,24,25,26,27,28,29,30,31,32,33,34,35,36,37,38].

### 3.2. Patient Characteristics

The 17 included studies consisted primarily of case reports and case series describing 114 patients diagnosed with PROS who received treatment with alpelisib. A substantial proportion of the study population (*n* = 57) was derived from the EPIK-P1 expanded-access study, representing the largest single dataset included in this analysis. Most of them were pediatric patients (78, 68.4%). The median age at treatment start was 12 years (range 0.3–68.0 years). Female-to-male ratio was 1.43. Patients presented with a wide spectrum of clinical manifestations, reflecting the heterogeneous nature of the disorder. The reported PROS phenotypes included congenital lipomatous overgrowth, vascular malformations, epidermal nevi, scoliosis/skeletal and spinal anomalies (CLOVES) syndrome (47, 41.2%), megalencephaly–capillary malformation (MCM) syndrome (13, 11.4%) and Klippel–Trenaunay syndrome (KTS) (11, 9.6%) (Table 1).

All the patients had documented postzygotic pathogenic variants in *PIK3CA*. Three variants—well known to be a mutational hotspot—were overall responsible for more than 50% of cases: c.3140A>G (p.His1047Arg) was reported in 23 patients (20%), c.1633G>A (p.Glu545Lys) in 22 patients (19%), and c.1624G>A (p.Glu542Lys) in 20 (17%). Prior treatment history was variably reported across studies. Among patients with available data, 63 had previously received sirolimus, with reported outcomes uniformly indicating partial clinical response or disease progression. Patients’ demographic characteristics and PROS subtypes are summarized in Table 1.

### 3.3. Clinical and Radiological Outcomes

The median length of treatment with alpelisib was 15 months (range 2.0–49.9 months). Dosing information was expressed as fixed daily doses, without specification of weight-adjusted (mg/kg) regimens. In pediatric patients, doses were most commonly around 50 mg/day, although higher doses (e.g., 125 mg/day) were occasionally reported. The lack of consistent weight-based dosing data precluded any meaningful analysis of dose–response relationships. Evaluating the most common PROS-related signs and symptoms reported before treatment initiation, clinical improvement in at least one symptom, such as pain, fatigue and motor impairment, was documented in 111 of 114 patients (97.3%). In two cases no information about clinical improvement was reported because they discontinued alpelisib before 3 months of treatment, and only one adult patient reported no clinical improvement. Imaging before and after treatment was reported in 60 cases (52.6%), corresponding to patients with available information on lesion volume change, either qualitatively or quantitatively.

Quantitative volumetric data were documented in 55 patients, while, in 5 cases, a reduction in lesion volume was described qualitatively without numerical assessment.

Among patients with quantitative data, 26 (47.3%) achieved a radiological response, defined as a reduction ≥20% in lesion volume, whereas 29 (52.7%) showed a reduction <20%. The mean ± SD change in sum of target lesion volume was −31.5 ± 21.4%. Specifically, only a subgroup of 23 patients had sufficiently detailed data to allow exploratory analyses to investigate potential factors associated with radiological response. No significant association was observed between hotspot *PIK3CA* mutations and response (*p* = 0.907), nor between patient age and volumetric change (*p* = 0.264), suggesting the absence of a clear genotype– or age–response correlation (Table 2).

Due to the limited variability in follow-up timing and the lack of standardized weight-adjusted dosing across studies, analyses exploring the relationship between treatment duration, dose, and radiological response were not pursued. Clinical improvement was observed even in all patients who did not demonstrate a measurable radiological response.

### 3.4. Safety and Adverse Events

Adverse events (AEs) reported after the first administration of alpelisib and up to 30 days after the last administration of alpelisib were variably reported across studies and overall experienced by 56.1% (*n* = 64) of patients.

The most common (incidence ≥10%) AEs of any grade were diarrhea (*n* = 14, 12.3%) and hyperglycemia (*n* = 14, 12.2), which is a known on-target effect of PI3K inhibition due to interference with insulin signaling.

Diarrhea was the most common AE in pediatric patients (10.3%; 8/78), whereas hyperglycemia was the most frequent in adults (27.7%; 10/36). Grade ≥ 3 AEs were reported in 17 patients (14.9%). Serious adverse events (SAEs) occurred in 22 patients (19.3% of the full study population). The most common SAE, reported only in adult patients, was hyperglycemia (*n* = 3, 2.3%). Hyperglycemia was significantly more frequent in adults compared to pediatric patients (*p* = 0.001), whereas no significant difference was observed for diarrhea (*p* = 0.512) (Table 3).

AEs resulting in dose reduction occurred in five adult patients (4.4% of the full study population) and two pediatric patients (1.8% of the full study population). Two patients permanently discontinued alpelisib because of an AE: an adult patient with hyperglycemic hyperosmolar coma and a pediatric patient with a growth suppression. Most of the patients (107/114, 93.9%) remained on treatment at the end of the reported follow-up. No deaths were reported.

## 4. Discussion

PROS represents a prototypical mosaic developmental disorder in which dysregulation of a central growth signaling pathway drives progressive tissue overgrowth [1,10,11]. The advent of targeted PI3K inhibitors has marked a major shift from symptomatic management toward mechanism-based therapy [12]. Despite the investigation of multiple strategies to target the PI3K–AKT–mTOR pathway and the development of several PI3K inhibitors, alpelisib has emerged over the past few years as the most widely used precision-medicine approach for PROS, owing to its selective inhibition of PI3Kα, favorable clinical efficacy and safety profile, and the growing body of clinical evidence supporting its use.

Real-world evidence in this setting remains limited, currently deriving mainly from compassionate use in case reports and small case series. At the same time, ongoing and recently completed clinical trials, including EPIK-P2, EPIK-P3, and EPIK-P4, are expected to provide more robust and standardized data on long-term efficacy, safety, and optimal dosing strategies.

In this review, we synthesized real-world clinical evidence from 114 patients with PROS treated with alpelisib, providing―to our knowledge―one of the largest aggregated datasets currently available on alpelisib therapy in PROS.

Overall, the available literature suggests that PI3K inhibition is associated with meaningful clinical improvement across a broad spectrum of PROS phenotypes [19,20,21]. Clinical benefit was observed in the vast majority of patients, including improvements in pain, fatigue, functional status, and cosmetic burden. These findings are consistent with results from the EPIK-P1 study [19].

These findings should be interpreted in the context of prior treatment exposure. A substantial proportion of patients had previously received sirolimus, with reported outcomes indicating only partial clinical response or disease progression prior to alpelisib initiation [34]. This observation underscores the limitations of mTOR inhibition in PROS and supports the rationale for targeting upstream components of the PI3K pathway as a more direct strategy to modulate disease-driving signaling.

A key finding of this review is the consistent discrepancy between clinical improvement and radiological response, representing a central observation of the present analysis. While clinical benefit was consistently reported in the literature, volumetric reduction of lesions was less unanimously documented. Importantly, clinical benefit was also observed in patients without a measurable radiological response, suggesting that symptom burden in PROS is not solely determined by lesion size but also by other factors. This discordance likely reflects the complex pathophysiology of PROS, in which vascular congestion, inflammatory processes and biomechanical effects contribute to clinical manifestations independently of volumetric changes [5].

The limited availability of standardized radiological outcomes, particularly in real-world settings, represents a major limitation. Although qualitative descriptions of “radiological improvement” were frequently provided, quantitative volumetric data were available only in a subset of patients, and only 23 cases had sufficiently detailed information to allow exploratory analyses [28,29,30,34], despite representing a primary endpoint in clinical trials. Within this limited subgroup, no significant association was observed between radiological response and either hotspot *PIK3CA* mutations or patient age, suggesting the absence of a clear genotype– or age–response relationship. However, these findings should be interpreted with caution given the small sample size and limited statistical power. Conversely, the frequent discrepancy between clinical improvement and radiological response suggests that lesion volume alone may not fully capture treatment benefit in PROS, underscoring the need for multidimensional outcome measures. The safety profile observed across reports appears broadly consistent with the known pharmacology of PI3K inhibition. Hyperglycemia and gastrointestinal toxicity represent the most frequently reported adverse events, with hyperglycemia occurring significantly more often in adult patients. Although most adverse events were manageable, serious adverse events were reported in a minority of cases, including rare but clinically relevant complications [24]. The consistency of safety findings between clinical trials and real-world reports further supports the safety profile of PI3K inhibition in PROS.

Nevertheless, important questions remain regarding long-term safety, particularly in pediatric populations who may require prolonged treatment. The long-term metabolic consequences of chronic PI3K inhibition, as well as its potential impact on growth and development, remain incompletely characterized.

Interpretation of the current evidence must consider several methodological limitations. Most available studies consist of case reports or small case series, inherently subject to selection bias and heterogeneous outcome reporting. The substantial heterogeneity in study design, outcome definitions, and follow-up duration further limits the strength and should be considered when interpreting these findings.

Radiological data were inconsistently reported and often lacked standardized volumetric assessment, limiting comparability across studies and reducing the robustness of aggregated estimates. In addition, the relationship between treatment duration, dose, and radiological response could not be meaningfully evaluated, as follow-up imaging was typically performed at a fixed time point (approximately 6 months after treatment initiation) and dosing was generally reported as fixed regimens rather than weight-adjusted values.

Furthermore, a potential overlap of patient populations across publications cannot be excluded, as the literature is largely derived from a limited number of specialized centers. Despite efforts to minimize this risk through cross-referencing demographic and genetic data, incomplete reporting may have limited the ability to identify duplicate cases. As a result, the effective sample size may be overestimated. In addition, more than half of the included patients were derived from a single study [19], which may have disproportionately influenced the overall findings and reduced the independence of the aggregated evidence.

Taken together, these findings highlight both the promise and the current limitations of PI3K inhibition in PROS. Future research should focus on the development of standardized outcome measures, particularly for radiological assessment, the identification of predictive biomarkers of response, and the implementation of prospective multicenter studies and international registries. Improved understanding of genotype–phenotype correlations and mosaic variant distribution may further refine patient selection and optimize therapeutic strategies. Finally, expanding the evaluation of PI3K inhibitors to other *PIK3CA*-driven vascular anomalies may further broaden the therapeutic landscape of these conditions [34,38].

## 5. Conclusions

Targeted PI3K inhibition with alpelisib represents a major advance in the treatment of PROS, shifting the therapeutic paradigm toward mechanism-based approaches. Real-world evidence supports its clinical benefit across a wide range of phenotypes, with a generally manageable safety profile. Nevertheless, the current evidence base remains limited by heterogeneous reporting and short follow-up. Future prospective studies and standardized outcome frameworks will be critical to define the long-term efficacy and safety of PI3K inhibition into clinical practice for PROS.

## Figures and Tables

**Table 1:** Demographic and clinical characteristics of patients with *PIK3CA*-related overgrowth spectrum (PROS) treated with alpelisib.

Characteristics	Pediatric Patients (<18 y) *N* = 78	Adult Patients (>18 y) *N* = 36	All Patients *N* = 114
Age, years			
Median (range)	7.5 (0.3–17.0)	42.0 (18–68.0)	12.0 (0.3–68.0)
Sex, *n* (%)			
Female	45 (57.7)	22 (61.1)	67
Male	33 (42.3)	14 (38.9)	47
PROS phenotypes			
CLOVES	32	15	47
MCM	13	0	13
KTS	5	6	11
HNLM	5	0	5
HFMH	5	0	5
FIL	4	0	4
CLAPO	1	0	1
HHML	1	0	1
Other	10	17	27

PROS, *PIK3CA*-related overgrowth spectrum; CLOVES, congenital lipomatous overgrowth, vascular malformations, epidermal nevi, and skeletal anomalies; MCM, megalencephaly–capillary malformation syndrome; KTS, Klippel–Trenaunay syndrome; HNLM, head and neck lymphatic malformations; HFMH, hemifacial myohyperplasia; FIL, fibroadipose-infiltrating lipomatosis; CLAPO, capillary malformation of the lower lip, lymphatic malformation of the face and neck, asymmetry, and partial/generalized overgrowth; HHML, hemihyperplasia with multiple lipomatosis. *N* represents total number of patients included in the analysis. *n* represents counts of patients.

**Table 2:** Association between clinical and genetic factors and radiological response.

		Δ < 20%	Δ ≥ 20%	*p*-Value
*PIK3CA* mutation	Non-hotspot	3	5	0.907
	Hotspot	6	9	
Age (years)	≤18 years	6	6	0.264
	>18 years	3	8	

Radiological response was defined as a Δ ≥ 20% reduction in lesion volume. Pediatric age was defined as ≤18 years. Hotspot mutations include H1047, E545, and E542 variants. No statistically significant associations were observed. The differences between groups were analyzed with Fisher’s exact test for categoric data.

**Table 3:** Comparison of most common adverse events between pediatric and adult patients. Adverse events are reported as counts and percentages. *p*-values were calculated using Fisher’s exact test.

Adverse Event		Yes	No	*p*-Value
Diarrhea	Pediatric *n* (%)	8 (10.3%)	70 (89.7%)	0.512
	Adult *n* (%)	6(16.7%)	30 (83.3%)	
Hyperglycemia	Pediatric *n* (%)	4 (5.1%)	74 (94.9%)	0.001
	Adult *n* (%)	10 (27.7%)	26 (72.3%)	

## Data Availability

No new data were created or analyzed in this study.

## Abbreviations

The following abbreviations are used in this manuscript:

PIK3CA	phosphatidylinositol-4,5-bisphosphate 3-kinase catalytic subunit alpha
PROS	PIK3CA-Related Overgrowth Spectrum
CLOVES	Congenital Lipomatous Overgrowth, Vascular malformations, Epidermal nevi, and Skeletal anomalies
MCAP	Megalencephaly–Capillary Malformation syndrome
KTS	Klippel–Trenaunay syndrome
HNLM	Head and Neck Lymphatic Malformations
HFMH	Hemifacial Myohyperplasia
FIL	Fibroadipose Infiltrating Lipomatosis
CLAPO	Capillary Malformation of the Lower Lip, Lymphatic Malformation of the Face and Neck, Asymmetry, and Partial/Generalized Overgrowth
HHML	Hemihyperplasia with Multiple Lipomatosis
